# The Impact of Social Participation on Health Outcomes Among Middle-Aged and Older Adults in China: Evidence from the Chinese General Social Survey

**DOI:** 10.3390/healthcare14131964

**Published:** 2026-07-02

**Authors:** Han Zhou, Hong Xu

**Affiliations:** School of Public Health, Chongqing Medical University, Chongqing 400016, China; 2024121798@stu.cqmu.edu.cn

**Keywords:** social participation, middle-aged and older adults, physical health, mental health, serial mediation

## Abstract

**Highlights:**

**What are the main findings?**

- Social participation was positively associated with both physical and mental health among middle-aged and older adults in China, with more consistent evidence observed for mental health.
- Loneliness and subjective well-being showed exploratory indirect associations linking social participation with health outcomes, accounting for a larger share of the association with mental health than with physical health.

**What are the implications of the main findings?**

- Promoting meaningful social participation may help support mental health and healthy aging among middle-aged and older adults.
- Community-based aging policies should emphasize not only participation frequency, but also participation quality, emotional connection, and sustained interpersonal interaction.

**Abstract:**

**Objective:** Against the background of rapid population aging, this study examined the associations between social participation and physical and mental health among middle-aged and older adults in China and explored potential psychosocial pathways involving loneliness and subjective well-being. **Methods:** Data were drawn from the 2023 Chinese General Social Survey (CGSS). A total of 3823 respondents aged 45 years and older were included in the analysis. Ordinary least squares regression models were used to examine the associations between social participation and physical and mental health. Sensitivity analyses, robustness checks, ordered logit models, and supplementary instrumental variable analyses were conducted to assess the consistency of the findings. Serial mediation analysis was used to explore the potential indirect associations involving loneliness and subjective well-being. **Results:** Higher levels of social participation were positively associated with better physical health and mental health among middle-aged and older adults (*p* < 0.001). Sensitivity and robustness analyses generally supported the consistency of these findings. Supplementary instrumental variable analyses showed a significant positive association with mental health, while the positive estimate for physical health was not statistically significant. Exploratory mediation analyses suggested that loneliness and subjective well-being may represent potential psychosocial pathways linking social participation to health outcomes. The total indirect association accounted for 22.40% of the total association with physical health and 33.24% of the total association with mental health. **Conclusions:** Social participation was positively associated with health among middle-aged and older adults in China, with more consistent evidence for mental health. Loneliness and subjective well-being may represent potential psychosocial pathways linking social participation to health outcomes.

## 1. Introduction

Population aging has emerged as a major public health challenge worldwide, with profound implications for health systems, social welfare, and population well-being. As the world’s largest developing country, China is experiencing particularly rapid population aging. According to data from the Seventh National Population Census, by the end of 2020, individuals in younger and middle-aged age groups accounted for 63.35% of the total population, representing a decline of 6.79 percentage points compared with the Sixth Census [1]. In contrast, the population aged 60 years and above reached approximately 260 million, accounting for 18.70% of the total population, an increase of 5.44 percentage points. These demographic trends indicate that China has moved beyond its initial phase of rapid population aging and is entering a period of more accelerated aging [2]. In this context, ensuring a healthy transition from middle age to older adulthood and improving health outcomes among older adults have become critical public health priorities.

The concept of social participation originates from activity theory in gerontology, which was proposed by the American sociologist Robert Havighurst in 1961. Activity theory posits that older adults can maintain psychological well-being and cope with role loss associated with aging by remaining engaged in active social roles, such as volunteering and paid employment. According to the World Health Organization (WHO), social participation refers to older adults’ involvement in society through activities such as volunteer services, community engagement, employment, lifelong learning, and cultural or recreational activities. In a broader sense, social participation encompasses both formal participation (e.g., organized volunteering) and informal participation (e.g., neighborhood mutual assistance) [3]. A growing body of evidence from both domestic and international studies has demonstrated that social participation is a key determinant of active aging among middle-aged and older adults. Numerous previous studies have reported that social participation is positively associated with a wide range of physical and mental health outcomes, including longevity [4], maintenance of activities of daily living [5], cognitive functioning [6], higher health-related quality of life [7], and the prevention of depression [8]. Activity theory suggests that participation in social activities helps meet older adults’ psychological and social needs, thereby buffering the negative effects of aging on mental health [9]. According to this theoretical perspective, older adults derive a sense of identity and meaning through engagement in social roles, which in turn adds vitality to their lives [10]. Moreover, regardless of older adults’ levels of physical activity, social participation has been shown to confer health benefits by providing opportunities to establish social networks and obtain adequate social support—one of the key protective factors against depression [11]. Empirical studies have further found that participation in volunteer activities among older adults is positively associated with both activities of daily living and instrumental activities of daily living, thereby improving individuals’ self-rated physical health [12]. Other scholars have reported that engagement in social activities can significantly reduce the risk of disability, with particularly pronounced protective effects on instrumental functional limitations [13]. Overall, individuals who are more actively engaged in social activities tend to exhibit better health outcomes than those with lower levels of participation. In addition, heterogeneity in individual characteristics—such as age, gender, income, and marital status—has been shown to significantly influence the health effects of social participation among older adults [14]. In relation to this, some studies have found that participation in multiple types of activities yields additional health benefits. Specifically, greater diversity in activity participation is associated with a lower likelihood of psychological symptoms among older adults. This finding is consistent with role accumulation theory, which posits that holding multiple social roles enhances access to social networks, resources, and self-esteem [15]. Taken together, these findings suggest that not only the frequency of social participation but also the diversity of activity types plays an important role in shaping health outcomes among older adults.

Against this background, this study used data from the 2023 Chinese General Social Survey (CGSS) to examine the association between social participation and health outcomes among middle-aged and older adults in China. Compared with previous studies, this study extends the existing literature in three ways. First, it measures social participation from the perspective of participation frequency, thereby capturing the intensity of social participation rather than merely distinguishing whether individuals participated in social activities. Second, it applies an instrumental variable approach as a supplementary analysis to account for potential endogeneity and improve the robustness of the estimates. Third, it constructs a serial mediation model involving loneliness and subjective well-being to explore potential psychosocial pathways linking social participation to health outcomes.

Based on previous theoretical and empirical evidence, this study proposed the following hypotheses.

**Hypothesis** **1.** *Higher levels of social participation are positively associated with better physical health among middle-aged and older adults.*

**Hypothesis** **2.** *Higher levels of social participation are positively associated with better mental health among middle-aged and older adults.*

**Hypothesis** **3.** *Loneliness and subjective well-being are potential psychosocial mediators in the association between social participation and health outcomes.*

**Hypothesis** **4.** *Social participation is indirectly associated with health outcomes through a serial pathway involving lower loneliness and higher subjective well-being.*

## 2. Data and Methods

### 2.1. Data Source

**Figure 1:**
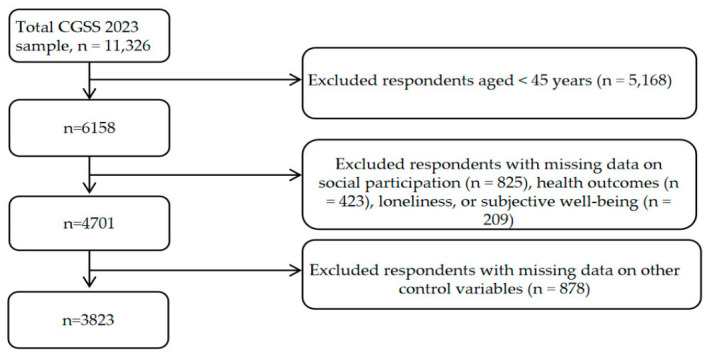
Flowchart of Sample Selection.

The Chinese General Social Survey (CGSS) is the earliest nationwide, comprehensive, and continuous academic survey project in China. It is administered by the National Survey Research Center at Renmin University of China. Following international survey standards, since 2003 the project has conducted annual cross-sectional surveys covering more than 10,000 households across provinces, municipalities, and autonomous regions in mainland China. The survey adopts a multistage, stratified probability proportional to size (PPS) sampling method. According to the 2023 survey design, a total of 100 county-level units and five major metropolitan areas were selected nationwide, covering 480 village/neighborhood committees and 12,000 individual respondents, and collecting data at multiple levels including society, community, household, and individual. The CGSS has been approved by the Ethics Committee of Renmin University of China, and all participants signed informed consent forms prior to the survey and voluntarily participated in the investigation. This study used the most recently released CGSS 2023 dataset. The original survey included 11,326 respondents. According to the study objective, respondents aged 45 years and older were first selected as the target middle-aged and older population, and 5168 respondents aged below 45 years were excluded. We then sequentially excluded respondents with missing data on social participation (n = 825), health outcome variables (n = 423), loneliness or subjective well-being (n = 209), and other control variables (n = 878). Finally, 3823 middle-aged and older respondents were included in the analytic sample. Missing data were handled using complete-case analysis. The sample selection process is shown in Figure 1.

### 2.2. Variable Measurement

#### 2.2.1. Dependent Variables

The dependent variables captured the health status of middle-aged and older adults across two dimensions: physical health and mental health. Physical health was assessed using the survey item “During the past four weeks, how often have health problems affected your work or other daily activities?” Responses were coded on a five-point Likert scale as follows: always = 1, often = 2, sometimes = 3, rarely = 4, and never = 5. Higher scores indicate fewer health-related limitations in daily activities and therefore better physical health. Mental health was measured using the item: “During the past four weeks, how often have you felt depressed or down?” Responses were coded as always = 1, often = 2, sometimes = 3, rarely = 4, and never = 5. Higher scores reflect fewer negative emotional experiences and better mental health.

#### 2.2.2. Independent Variable

Social participation refers to the process and extent to which individuals interact, cooperate, contribute, and assume social roles in relation to other people, groups, organizations, or communities. In this study, social participation was measured using relevant items from the CGSS questionnaire, which asked respondents whether they had frequently engaged in the following activities during their leisure time in the past year. A total of 12 activities were included: watching television or videos, going out to watch movies, shopping, reading books, newspapers, or magazines, participating in cultural activities such as attending concerts, performances, or exhibitions, gathering with relatives who do not live together, gathering with friends, listening to music at home, participating in physical exercise, watching sports events, doing handicrafts, and using the Internet.

It should be noted that this study adopted a broad operationalization of social participation, aiming to capture respondents’ overall level of activity engagement in daily life and social contexts. Considering that different activities vary in the degree of social interaction involved, we further classified them into three categories according to their interactive attributes. The first category was socially interactive activities, including gathering with relatives who do not live together and gathering with friends. The second category was public-outing and cultural leisure activities, including going out to watch movies, shopping, participating in cultural activities, participating in physical exercise, and watching sports events. The third category was individualized or general leisure activities, including watching television or videos, reading books, newspapers, or magazines, listening to music at home, doing handicrafts, and using the Internet. In subsequent analyses, in addition to using the overall social participation index, activity-type-based sensitivity analyses were conducted to examine whether the findings were affected by the operationalization of social participation.

Response options for each activity were coded as never = 1, several times a year or less = 2, several times a month = 3, several times a week = 4, and every day = 5. The overall social participation index was constructed by summing the scores of the 12 activity items, yielding a possible range from 12 to 60. All items were equally weighted, and no reverse coding was applied. Higher scores indicate more frequent engagement in leisure and social activities and therefore a higher overall level of social participation. For the activity-type-based sensitivity analyses, each activity-type index was calculated as the average score of the activities included in that category, with possible scores ranging from 1 to 5. Higher scores on each activity-type index indicate more frequent participation in that specific type of activity. In the present study, the internal consistency of the social participation scale was acceptable (Cronbach’s alpha = 0.677). The Kaiser–Meyer–Olkin (KMO) value was 0.802, indicating that the data were suitable for factor analysis. In addition, the first factor explained 25.652% of the total variance, which is below the commonly used threshold of 40%, suggesting that common method bias was unlikely to be a major concern.

#### 2.2.3. Mediating Variables

Two psychosocial variables, loneliness and subjective well-being, were included as mediators. Loneliness was measured using the Chinese version of the three-item UCLA Loneliness Scale (UCLA-3) [16]. Respondents were asked how frequently, during the past four weeks, they felt “a lack of companionship,” “left out,” and “isolated from others.” The three items were averaged to generate a loneliness score ranging from 1 to 5, with higher scores indicating greater loneliness. The Cronbach’s alpha coefficient for this scale in the present study was 0.790, indicating good internal consistency. Subjective well-being was assessed using the item “Overall, how happy do you feel about your life?” Response options ranged from very unhappy to very happy and were coded from 1 to 5, with higher scores indicating greater subjective well-being.

#### 2.2.4. Control Variables

Based on the previous literature [17,18], we included a set of control variables associated with health outcomes among middle-aged and older adults, including gender, age, marital status, educational attainment, place of residence, and pension insurance coverage. In addition, self-rated health was used as a proxy outcome in the robustness analysis. Self-rated health was measured by the question “How would you rate your current health?” Responses were coded as very unhealthy = 1, relatively unhealthy = 2, fair = 3, relatively healthy = 4, and very healthy = 5. Higher scores indicate better self-perceived health status.

### 2.3. Statistical Analysis

All statistical analyses were conducted using SPSS version 24.0. Missing data were handled using complete-case analysis. Descriptive statistics were used to summarize participant characteristics. Categorical variables were presented as frequencies and percentages, and continuous variables as means and standard deviations.

Ordinary least squares (OLS) regression models were first used to examine the associations between social participation and physical and mental health, adjusting for gender, age, education, residence, pension insurance, and marital status. To assess whether the findings were affected by the broad operationalization of social participation, activity-type-based sensitivity analyses were conducted by replacing the overall social participation index with three activity-type indices.

Several robustness checks were then performed, including using self-rated health as an alternative outcome, excluding respondents aged 85 years and older, and estimating ordered logit models for the ordered health outcomes. To further account for potential endogeneity, an instrumental variable analysis was conducted using two-stage least squares estimation, with participation in the most recent neighborhood/village committee election as the instrument for social participation. Finally, serial mediation analysis was conducted using the PROCESS macro for SPSS to examine whether loneliness and subjective well-being served as potential psychosocial pathways linking social participation to health outcomes. The models controlled for gender, age, educational attainment, residence, pension insurance coverage, and marital status. Indirect effects were tested using 5000 bootstrap resamples, with statistical significance determined by 95% confidence intervals that did not include zero. Given the cross-sectional design, the mediation results were interpreted as exploratory indirect associations rather than causal mediation effects. A two-sided *p* < 0.05 was considered statistically significant.

## 3. Results

### 3.1. General Characteristics of the Study Participants

A total of 3823 participants from the CGSS 2023 survey were included in this study. The basic characteristics of the sample are presented in Table 1. As the study focused on the middle-aged and older adults in China, all respondents were aged 45 years or older, with a range from 45 to 94 years and a mean age of 62.37 ± 10.26 years. Males accounted for 46.14% of the sample and females 53.86%. The majority of participants (79.36%) were married (with a spouse), while 20.64% were without a spouse. In terms of educational attainment, 14.13% had received no formal education; most respondents had completed primary school (30.39%) or junior high school (29.84%); 17.84% had senior high school education, and only 7.79% held a college degree or above. Regarding residence, 60.16% lived in towns or rural areas, and 39.84% resided in urban areas. With respect to pension coverage, 82.92% of the middle-aged and older adults had pension insurance, whereas 17.08% had no pension coverage.

### 3.2. Baseline Regression Results for the Association Between Social Participation and Health Outcomes

The baseline regression results for the associations between social participation and health outcomes among middle-aged and older adults are presented in Table 2. Social participation was positively associated with physical health (β = 0.433, *p* < 0.001), suggesting that higher social participation was related to better physical health scores. Gender, age, and education were also significantly associated with physical health (*p* < 0.01). Social participation was likewise positively associated with mental health (β = 0.364, *p* < 0.001), indicating that higher social participation was related to better mental health. In addition, gender, education, residence, and marital status were significantly associated with mental health outcomes (*p* < 0.05).

### 3.3. Activity-Type-Based Sensitivity Analysis

To further assess whether the baseline findings were affected by the broad measurement of social participation, we conducted an activity-type-based sensitivity analysis. The results are presented in Table 3. For physical health, the overall social participation index was positively associated with physical health in Model 2 (β = 0.433, *p* < 0.001). When social participation was disaggregated into three activity types in Model 3, socially interactive activities (β = 0.134, *p* < 0.001), public-outing and cultural leisure activities (β = 0.357, *p* < 0.001), and individualized or general leisure activities (β = 0.202, *p* < 0.001) were all positively associated with physical health. The explanatory power of the model remained largely stable after the activity-type variables were introduced.

A similar pattern was observed for mental health in Table 4. The overall social participation index was positively associated with mental health in Model 2 (β = 0.364, *p* < 0.001). In Model 3, socially interactive activities (β = 0.206, *p* < 0.001), public-outing and cultural leisure activities (β = 0.184, *p* < 0.001), and individualized or general leisure activities (β = 0.231, *p* < 0.001) were all significantly and positively associated with mental health. These findings suggest that the positive associations between social participation and health outcomes were generally robust after distinguishing different types of activities. They also indicate that the overall social participation index may capture both interpersonal engagement and broader activity engagement in daily life.

### 3.4. Robustness Analysis

To assess the robustness of the baseline estimates, additional analyses were conducted using three approaches: substituting the dependent variable, restricting the study sample, and estimating ordered logit models. The results are presented in Table 5. First, when self-rated health was used as an alternative outcome, social participation remained positively associated with better self-rated health (β = 0.386, *p* < 0.001), consistent with the baseline findings. Second, following common practice in geriatric epidemiology [19], individuals aged 85 years and older were excluded, and the OLS models were re-estimated using the restricted sample aged 45–84 years. In this subsample, social participation remained significantly associated with both physical health (β = 0.431, *p* < 0.001) and mental health (β = 0.368, *p* < 0.001).

Third, because the physical and mental health outcomes were measured using five-point ordered response categories, ordered logit models were further estimated as supplementary robustness checks. The results showed that social participation remained positively associated with higher levels of physical health (β = 0.599, *p* < 0.001) and mental health (β = 0.592, *p* < 0.001), which was broadly consistent with the baseline OLS estimates. However, the parallel-lines tests were statistically significant, indicating that the proportional odds assumption was not fully satisfied. Therefore, the ordered logit results should be interpreted as supplementary evidence rather than as the primary model estimates. Overall, these findings suggest that the positive associations between social participation and health outcomes were generally robust across alternative outcome measurement, sample restriction, and model specification.

### 3.5. Instrumental Variable Analysis of the Association Between Social Participation and Health Outcomes

To address potential endogeneity arising from reverse causality and omitted variables, this study further employed an instrumental variable approach. Specifically, whether respondents participated in the most recent neighborhood committee or village committee election was used as an instrument for social participation. This variable reflects individuals’ tendency to engage in community public affairs and is therefore theoretically related to their level of social participation. However, given that election participation may also be associated with socioeconomic status, mobility, cognitive functioning, social capital, or broader community engagement, the exclusion restriction cannot be fully verified. Therefore, the instrumental variable analysis in this study was used primarily as a supplementary robustness check rather than as definitive evidence of causal identification. The results of the instrumental variable analysis are presented in Table 6.

The analysis focused mainly on the relevance of the instrument and the potential weak-instrument problem. The first-stage F-statistic was 198.106, which was far above the conventional threshold of 10, suggesting that weak-instrument bias was unlikely. In the first-stage regression, participation in neighborhood or village committee election voting was significantly associated with social participation (coefficient = −0.151, standard error = 0.016, *p* < 0.001), indicating a strong statistical association between the instrument and the potentially endogenous explanatory variable. It should be noted that the direction of this coefficient should be interpreted in light of the coding scheme of the voting variable.

In the second-stage regression, the predicted value of social participation obtained from the instrument was used to replace the original social participation variable. The two-stage least squares (2SLS) results showed that the association between social participation and mental health remained positive and statistically significant, and the estimated coefficient was larger than that obtained from the OLS model. This pattern suggests that the supplementary instrumental variable (IV) estimate was consistent with a positive association, further supporting the robustness of the main findings. By contrast, the estimated association between social participation and physical health remained positive but was not statistically significant in the 2SLS model, suggesting that the evidence for physical health was weaker and should be interpreted more cautiously. Overall, the instrumental variable results provide supplementary support for the robustness of the association between social participation and mental health, whereas the corresponding evidence for physical health is less conclusive.

### 3.6. Serial Mediation Analysis of the Association Between Social Participation and Health Outcomes

The analyses showed significant indirect associations involving loneliness and subjective well-being in the relationship between social participation and health outcomes (*p* < 0.001). The corresponding regression results are presented in Table 7.

The bootstrap mediation results for physical health are presented in Table 8. The total association between social participation and physical health was 0.433 (95% CI: 0.353, 0.512). The direct association was 0.336 (95% CI: 0.258, 0.415). The indirect association through loneliness was 0.028 (95% CI: 0.013, 0.043), accounting for 6.47% of the total association. The indirect association through subjective well-being was 0.064 (95% CI: 0.046, 0.084), accounting for 14.78% of the total association. The serial indirect association through loneliness and then subjective well-being was 0.005 (95% CI: 0.002, 0.008), accounting for 1.15% of the total association. The total indirect association was 0.096, representing 22.40% of the total association.

The bootstrap mediation results for mental health are presented in Table 9. The total association between social participation and mental health was 0.364 (95% CI: 0.292, 0.437). The direct association was 0.243 (95% CI: 0.174, 0.312). The indirect association through loneliness was 0.037 (95% CI: 0.010, 0.049), accounting for 10.16% of the total association. The indirect association through subjective well-being was 0.078 (95% CI: 0.059, 0.099), accounting for 21.29% of the total association. The serial indirect association through loneliness and then subjective well-being was 0.006 (95% CI: 0.003, 0.009), accounting for 1.65% of the total association. The total indirect association was 0.121, representing 33.24% of the total association.

## 4. Discussion

### 4.1. Social Participation and Physical Health: Interpretation and Implications

The findings of this study suggest that social participation was positively associated with physical health among middle-aged and older adults in China. However, after accounting for potential endogeneity through an instrumental variable approach, the estimate was attenuated and no longer statistically significant. This pattern suggests that the relationship between social participation and physical health may not operate primarily through short-term or direct physiological pathways, but may instead reflect longer-term behavioral and environmental processes [20]. This interpretation is consistent with the broader public health context, in which physical health among middle-aged and older adults is shaped by chronic disease management, lifestyle-related risk factors, and marked urban–rural and socioeconomic disparities [21]. Social participation may be linked to better physical health by increasing daily activity, supporting more regular routines, and enhancing health awareness [22]. At the same time, these associations may depend on sustained engagement and may vary according to baseline health, living environment, and access to social and health-related resources [23]. Accordingly, the attenuation observed after the supplementary instrumental variable specification does not necessarily imply that social participation is unimportant for physical health. Rather, it highlights the complexity of this relationship. From a policy perspective, simply encouraging participation may be insufficient to yield short-term improvements in physical health. Social participation initiatives may be more effective when combined with primary public health services, community-based health management, and chronic disease prevention efforts. Embedding health screening, exercise guidance, and health education into community activities may help make participation more health-supportive.

### 4.2. Social Participation and Mental Health: Interpretation and Implications

Compared with physical health, the association between social participation and mental health appeared more stable and consistent across the analyses. The supplementary instrumental variable analysis also yielded results consistent with a positive relationship between social participation and mental health, further supporting the robustness of the main findings. This finding is particularly meaningful in the context of ongoing social change in China. Changes such as smaller family size, reduced intergenerational co-residence, and widespread retirement have contributed to a contraction of social roles and emotional support among middle-aged and older adults [24]. Mental health concerns—including depression, loneliness, and low mood—have therefore become increasingly important, yet they often remain underrecognized in later life [25]. In this context, social participation may serve not only as a leisure-related behavior but also as a way for older adults to maintain social identity, affirm self-worth, and sustain emotional connection [26]. The present findings show a stronger association with mental health than with physical health, suggesting that mental health may be particularly sensitive to the social environment. This also implies that expanding opportunities for meaningful participation may offer mental health benefits at relatively low institutional cost [27]. Within active aging policy frameworks, social participation therefore merits greater attention as a component of mental health promotion, especially for groups at higher risk of poor mental health, such as widowed individuals, those with lower educational attainment, and older adults living in rural areas [28].

### 4.3. Potential Psychosocial Pathways Linking Social Participation and Health

The serial mediation analyses provided exploratory evidence that loneliness and subjective well-being may help explain the association between social participation and health outcomes among middle-aged and older adults. However, because the data were cross-sectional, the assumed temporal sequence among social participation, loneliness, subjective well-being, and health cannot be empirically established. Therefore, these results should be interpreted as indirect statistical associations rather than as evidence of a confirmed causal mediation mechanism. Within this limitation, the findings suggest that lower loneliness and higher subjective well-being may constitute plausible psychosocial pathways linking social participation to better health. The indirect associations accounted for a larger proportion of the total association with mental health than with physical health, indicating that psychosocial factors may be particularly relevant to mental health outcomes. More specifically, loneliness appeared to be closely related to the association between social participation and health. Among middle-aged and older adults in China, loneliness may not be determined solely by objective living arrangements, such as living alone, but may instead be more strongly shaped by the quality of social interaction and emotional connection [29]. Social participation may provide opportunities for interpersonal contact and social role engagement, which may be associated with lower perceived loneliness [30]. Lower loneliness may, in turn, be linked to higher subjective well-being, more positive life evaluation, and better emotional status [31].

These findings have implications for the design of community-based aging policies, although they should be interpreted cautiously. In some settings, efforts to promote social participation among older adults have emphasized formal or symbolic participation, such as organizing activities or expanding venues, while paying insufficient attention to participants’ emotional experiences and psychological gains. The present findings suggest that participation may be more strongly associated with health when it involves sustained interaction, emotional connection, and subjective meaning. Future community programs may therefore benefit from placing greater emphasis on the interactive and emotionally engaging aspects of participation. Small, stable interest groups, mutual-aid activities, and structured volunteer programs may be especially helpful in strengthening social connection and improving subjective well-being among middle-aged and older adults [32].

### 4.4. Limitations

This study has several limitations. First, the cross-sectional design limits causal interpretation. Although an instrumental variable approach was used to account for potential endogeneity, the analyses cannot fully rule out omitted variable bias, reverse causality, or reciprocal relationships between social participation and health. In particular, individuals with better health or higher subjective well-being may be more likely to participate in social activities. Therefore, the findings should be interpreted primarily as evidence of associations rather than definitive causal effects. Future studies using longitudinal data or quasi-experimental designs are needed to clarify the temporal order and longer-term health relevance of social participation.

Second, the measurement of social participation has limitations. This study adopted a broad operationalization of social participation that included cultural, recreational, interpersonal, and daily leisure activities. Although this approach helps capture respondents’ overall level of activity engagement, some activities may involve limited direct social interaction. The activity-type-based sensitivity analysis partly addressed this issue, but the construct validity of the social participation measure remains an important consideration. Future research should distinguish more clearly between socially interactive activities, public or community-based activities, and individualized leisure activities, and should also examine participation quality, interaction depth, and emotional engagement.

Third, the health outcomes were measured using single self-reported items. The physical health item mainly reflected health-related limitations in daily activities, whereas the mental health item captured the frequency of feeling depressed or down during the previous four weeks. These measures do not fully represent multidimensional physical or mental health status and may be influenced by subjective perception or current emotional state. In addition, subjective well-being and mental health may have some conceptual overlap, which should be considered when interpreting the mediation results. Future studies could incorporate objective health indicators, clinical measures, or validated multidimensional scales to improve measurement precision.

Fourth, although several demographic and socioeconomic covariates were included, residual confounding may still exist. Factors such as income, employment or retirement status, chronic disease burden, disability, activities of daily living, living arrangements, social support, health behaviors, and regional characteristics may influence both social participation and health outcomes. The omission of these variables may affect the estimated associations. In addition, complete-case analysis may introduce potential selection bias if respondents with missing data differ systematically from those included in the final sample.

Finally, although the CGSS adopts a complex sampling design, the present analysis did not fully incorporate sampling weights, clustering, or stratification. This may limit the generalizability of the estimates. Moreover, the instrumental variable used in this study—participation in neighborhood or village committee election voting—showed strong statistical relevance, but its exclusion restriction cannot be fully verified. Voting participation may also be related to socioeconomic status, mobility, cognitive functioning, social capital, or broader community engagement. Therefore, the instrumental variable results should be regarded as supplementary robustness evidence rather than definitive causal identification.

## 5. Conclusions

Using data from the 2023 Chinese General Social Survey, this study found that social participation was positively associated with both physical and mental health among middle-aged and older adults in China. The findings were generally robust across several supplementary analyses, although the evidence was more consistent for mental health than for physical health. The exploratory mediation analyses further suggested that loneliness and subjective well-being may represent potential psychosocial pathways linking social participation to health outcomes, particularly mental health. These findings suggest that community-based aging policies should not only increase opportunities for participation but also improve the quality, continuity, and emotional meaningfulness of social engagement. programs that promote sustained interpersonal interaction, reduce loneliness, and enhance subjective well-being may be especially valuable for supporting healthy aging. Given the cross-sectional design and measurement limitations, the findings should be interpreted as associations rather than definitive causal effects.

## Figures and Tables

**Table 1:** Characteristics of the Middle-Aged and Older Adults.

Variable	Category	Frequency/Mean	Percentage (%)/SD
Gender	Male	1764	46.14
	Female	2059	53.86
Age (years)	45–59	1651	43.18
	60–94	2172	56.82
Marital Status	Without spouse	789	20.64
	Married (with spouse)	3034	79.36
Education	No formal education	540	14.13
	Primary school	1162	30.39
	Junior high school	1141	29.84
	Senior high school	682	17.84
	College or above	298	7.79
Residence	Urban	1523	39.84
	Town or rural	2300	60.16
Pension Insurance	No	653	17.08
	Yes	3170	82.92
Loneliness score	-	1.58	0.79
Subjective well-being	-	3.87	0.88
Self-rated health	-	3.14	1.14

Note: Data are presented as frequency (percentage) for categorical variables and mean (standard deviation, SD) for continuous variables (loneliness, subjective well-being, and self-rated health scores).

**Table 2:** Baseline Regression Results for the Association Between Social Participation and the Health Outcomes of Middle-Aged and Older Adults.

Variable	Physical Health	Mental Health
Social participation	0.433 *** (0.041)	0.364 *** (0.037)
Gender (Male = 1, Female = 2)	−0.129 ** (0.041)	−0.141 *** (0.037)
Age (≥45 years)	−0.019 *** (0.002)	0.000 (0.002)
Education (Illiterate = 1, Primary–College = 2–5)	0.091 *** (0.021)	0.104 *** (0.019)
Residence (Urban = 1, Rural = 2)	−0.329 *** (0.046)	−0.220 *** (0.042)
Pension insurance (No = 1, Yes = 2)	0.054 (0.053)	−0.083 (0.048)
Marital status (Without spouse = 1, With spouse = 2)	0.042 (0.051)	0.110 * (0.046)
Constant	4.098 *** (0.258)	3.214 *** (0.235)
R-squared	0.128	0.089
Adjusted R-squared	0.127	0.087
F-statistic	80.358 ***	53.262 ***
N	3823	3823

Note: Standard errors are reported in parentheses. ***, **, and * denote significance at the 1%, 5%, and 10% levels, respectively.

**Table 3:** Activity-Type-Based Sensitivity Analysis of the Association Between Social Participation and Physical Health.

Variable	Model 1	Model 2	Model 3
Gender (Male = 1, Female = 2)	−0.119 ** (0.041)	−0.129 ** (0.041)	−0.125 ** (0.041)
Age (≥45 years)	−0.021 *** (0.002)	−0.019 *** (0.002)	−0.019 *** (0.002)
Education (Illiterate = 1, Primary–College = 2–5)	0.171 *** (0.020)	0.091 *** (0.021)	0.090 *** (0.021)
Residence (Urban = 1, Rural = 2)	−0.417 *** (0.046)	−0.329 *** (0.046)	−0.324 *** (0.046)
Pension insurance (No = 1, Yes = 2)	0.085 (0.054)	0.054 (0.053)	0.053 (0.053)
Marital status (Without spouse = 1, With spouse = 2)	0.053 (0.052)	0.042 (0.051)	0.042 (0.051)
Social participation		0.433 *** (0.041)	
Socially interactive activities			0.134 *** (0.028)
Public-outing and cultural leisure activities			0.357 *** (0.039)
Individualized or general leisure activities			0.202 *** (0.031)
Constant	4.994 *** (0.248)	4.098 *** (0.258)	4.086 *** (0.260)
R-squared	0.103	0.128	0.129
Adjusted R-squared	0.101	0.127	0.127
F-statistic	72.692 ***	80.358 ***	62.960 ***
N	3823	3823	3823

Note: Standard errors are reported in parentheses. Model 1 included only control variables. Model 2 added the overall social participation index. Model 3 replaced the overall social participation index with three activity-type indices. All models controlled for gender, age, education, residence, pension insurance, and marital status. *** and ** denote significance at the 1% and 5% levels, respectively.

**Table 4:** Activity-Type-Based Sensitivity Analysis of the Association Between Social Participation and Mental Health.

Variable	Model 1	Model 2	Model 3
Gender (Male = 1, Female = 2)	−0.133 *** (0.038)	−0.141 *** (0.037)	−0.133 *** (0.037)
Age (≥45 years)	−0.001 (0.002)	0.000 (0.002)	0.001 (0.002)
Education (Illiterate = 1, Primary–College = 2–5)	0.172 *** (0.018)	0.104 *** (0.019)	0.101 *** (0.019)
Residence (Urban = 1, Rural = 2)	−0.294 *** (0.042)	−0.220 *** (0.042)	−0.212 *** (0.042)
Pension insurance (No = 1, Yes = 2)	−0.057 (0.049)	−0.083 (0.048)	−0.085 (0.048)
Marital status (Without spouse = 1, With spouse = 2)	0.119 * (0.047)	0.110 * (0.046)	0.108 * (0.046)
Social participation		0.364 *** (0.037)	
Socially interactive activities			0.206 *** (0.025)
Public-outing and cultural leisure activities			0.184 *** (0.035)
Individualized or general leisure activities			0.231 *** (0.028)
Constant	3.969 *** (0.225)	3.214 *** (0.235)	3.216 *** (0.236)
R-squared	0.066	0.089	0.092
Adjusted R-squared	0.064	0.087	0.090
F-statistic	44.850 ***	53.262 ***	43.121 ***
N	3823	3823	3823

Note: Standard errors are reported in parentheses. Model 1 included only control variables. Model 2 added the overall social participation index. Model 3 replaced the overall social participation index with three activity-type indices. All models controlled for gender, age, education, residence, pension insurance, and marital status. *** and * denote significance at the 1%, and 10% levels, respectively.

**Table 5:** Robustness Checks for the Association Between Social Participation and Health Outcomes.

	Alternative Outcome	Restricted Sample		Ordered Logit Model	
Variable	Self-Rated Health	Physical Health	Mental Health	Physical Health	Mental Health
Social participation	0.386 *** (0.036)	0.431 *** (0.041)	0.368 *** (0.037)	0.599 *** (0.061)	0.592 *** (0.062)
Control variables	Yes	Yes	Yes	Yes	Yes
N	3823	3766	3766	3823	3823
Parallel-lines test	—	—	—	*p* < 0.001	*p* < 0.001

Note: Standard errors are reported in parentheses. All models controlled for gender, age, education, residence, pension insurance, and marital status. *** *p* < 0.001. The ordered logit models were estimated as supplementary robustness checks because the health outcomes were measured using ordered response categories.

**Table 6:** Instrumental Variable Estimates for the Association Between Social Participation and Health Outcomes.

Variable	OLS		2SLS		
			First Stage	Second Stage	
	Physical Health	Mental Health		Physical Health	Mental Health
Instrument			−0.151 *** (0.016)		
Social Participation	0.433 *** (0.041)	0.364 *** (0.037)		0.450 (0.279)	0.934 ** (0.253)
Control variables	Yes	Yes	Yes	Yes	Yes
Observations	3823	3823	3823	3823	3823
Constant	4.098 *** (0.258)	3.214 *** (0.235)	2.556 *** (0.101)	4.173 *** (0.675)	1.616 *** (0.611)

Note: Robust standard errors are reported in parentheses. *** and ** denote significance at the 1% and 5% levels, respectively. All models control for gender, age, education, residence, pension insurance, and marital status.

**Table 7:** Regression Results for the Serial Mediation Analysis.

Dependent Variable	Independent Variable(s)	R	R2	F	β	t
Loneliness	Social participation	0.299 ***	0.089	53.510 ***	−0.100 ***	−3.939
Subjective well-being	Social participation	0.314 ***	0.098	52.004 ***	0.294 ***	10.530
	Loneliness				−0.217 ***	−12.206
Physical Health	Social participation	0.428 ***	0.184	95.239	0.336 ***	8.415
	Loneliness				−0.277 ***	−10.840
	Subjective well-being				0.218 ***	9.526
Mental Health	Social participation	0.454 ***	0.206	109.730	0.243 ***	6.917
	Loneliness				−0.376 ***	−16.688
	Subjective well-being				0.266 ***	13.230

Note: *** denotes significance at the 1% level.

**Table 8:** Serial Mediation Analysis for the Association Between Social Participation and Physical Health.

Model Path	Effect (95% CI)	Boot SE	Proportion of Total Effect (%)
Total effect	0.433 (0.353, 0.512)	0.041	100.00
Direct effect	0.336 (0.258, 0.415)	0.040	77.60
Total indirect effect	0.096 (0.072, 0.122)	0.013	22.40
Path 1	0.028 (0.013, 0.043)	0.007	6.47
Path 2	0.064 (0.046, 0.084)	0.010	14.78
Path 3	0.005 (0.002, 0.008)	0.001	1.15

Note: Path 1: Social participation → Loneliness → Physical health; Path 2: Social participation → Subjective well-being → Physical health; Path 3: Social participation → Loneliness → Subjective well-being → Physical health. CI = confidence interval; Boot SE = bootstrap standard error.

**Table 9:** Serial Mediation Analysis for the Association Between Social Participation and Mental Health.

Model Path	Effect (95% CI)	Boot SE	Proportion of Total Effect (%)
Total effect	0.364 (0.292, 0.437)	0.037	100.00
Direct effect	0.243 (0.174, 0.312)	0.035	66.76
Total indirect effect	0.121 (0.093, 0.152)	0.015	33.24
Path 1	0.037 (0.010, 0.049)	0.010	10.16
Path 2	0.078 (0.059, 0.099)	0.010	21.29
Path 3	0.006 (0.003, 0.009)	0.002	1.65

Note: Path 1: Social participation → Loneliness → Mental health; Path 2: Social participation → Subjective well-being → Mental health; Path 3: Social participation → Loneliness → Subjective well-being → Mental health. CI = confidence interval; Boot SE = bootstrap standard error.

## Data Availability

The data presented in this study are available on request from the corresponding author due to privacy and ethical restrictions. The data are not publicly available because they contain participant-related information and public sharing was not included in the informed consent.

## References

[1] Tu W., Zeng X., Liu Q. (2022). Aging tsunami coming: The main finding from China’s seventh national population census. Aging Clin. Exp. Res..

[2] Fang E.F., Fang Y., Chen G., Wang H.-L., Zhang J., Wu C., Liao J., Xie C., Liu X., Wang K. (2025). Adapting health, economic and social policies to address population aging in China. Nat. Aging.

[3] Kalache A., Gatti A. (2003). Active ageing: A policy framework. Adv. Gerontol. Uspekhi Gerontol..

[4] Avlund K., Lund R., Holstein B.E., Due P., Sakari-Rantala R., Heikkinen R.-L. (2004). The impact of structural and functional characteristics of social relations as determinants of functional decline. J. Gerontol. Ser. B Psychol. Sci. Soc. Sci..

[5] Kanamori S., Kai Y., Aida J., Kondo K., Kawachi I., Hirai H., Shirai K., Ishikawa Y., Suzuki K., The JAGES Group (2014). Social participation and the prevention of functional disability in older Japanese: The JAGES cohort study. PLoS ONE.

[6] Lee S.H., Kim Y.B. (2016). Which type of social activities may reduce cognitive decline in the elderly?: A longitudinal population-based study. BMC Geriatr..

[7] Park H.K., Chun S.Y., Choi Y., Lee S.Y., Kim S.J., Park E.-C. (2015). Effects of social activity on health-related quality of life according to age and gender: An observational study. Health Qual. Life Outcomes.

[8] Takagi D., Kondo K., Kawachi I. (2013). Social participation and mental health: Moderating effects of gender, social role and rurality. BMC Public Health.

[9] Blazer D.G. (1982). Social support and mortality in an elderly community population. Am. J. Epidemiol..

[10] Ryff C.D. (1982). Successful aging: A developmental approach. Gerontologist.

[11] Glass T.A., De Leon C.F.M., Bassuk S.S., Berkman L.F. (2006). Social engagement and depressive symptoms in late life: Longitudinal findings. J. Aging Health.

[12] Gao X., Wen X. (2019). The impact of urban elderly volunteer service participation on their health. Popul. Econ..

[13] Hu H., Li Y., Zhang C., Zhang J. (2017). Participation of social activities, health promotion and disability prevention: Empirical analysis based on active ageing structure. Chin. J. Popul. Sci..

[14] Liu H., Yan H., Li R., Xu S., Xu M. (2022). Depression status and its influencing factors in urban and rural elderly in China. Chin. J. Hosp. Stat..

[15] Sieber S.D. (1974). Toward a theory of role accumulation. Am. Sociol. Rev..

[16] Hughes M.E., Waite L.J., Hawkley L.C., Cacioppo J.T. (2004). A short scale for measuring loneliness in large surveys: Results from two population-based studies. Res. Aging.

[17] Gao Y., Chen L., Jia Z., Zhao L., Yang Y., Liu C. (2024). Social participation and health in middle-aged and older empty nesters: A study on gender differences. SSM-Popul. Health.

[18] Jing M., Wang Q., Jia Y., Yu X., Tian K. (2025). The impact of social participation on mental health among the older adult in China: An analysis based on the mental frailty index. Front. Public Health.

[19] Fitzsimmons P., Blayney S., Mina-Corkill S., Scott G. (2012). Older participants are frequently excluded from Parkinson’s disease research. Park. Relat. Disord..

[20] Abe T., Seino S., Tomine Y., Nishi M., Hata T., Shinkai S., Fujiwara Y., Kitamura A. (2022). Identifying the specific associations between participation in social activities and healthy lifestyle behaviours in older adults. Maturitas.

[21] Cui J., Chai X., Ye L., Shao R., Shi X., Lv Y., Zhang J. (2024). Exploring important health problems for Chinese older adults based on Delphi method. Chin. J. Dis. Control.

[22] Wan Y.Y., Zeng Y.B., Fang Y. (2021). A study on the effects of labor participation on the health of the retired elderly in China. Chin. J. Health Policy.

[23] Levasseur M., Naud D., Bruneau J.-F., Généreux M. (2020). Environmental characteristics associated with older adults’ social participation: The contribution of sociodemography and transportation in metropolitan, urban, and rural areas. Int. J. Environ. Res. Public Health.

[24] Lei Y., Lao J., Liu J. (2022). Participation in community seniors’ organizations and mental health among retired adults in urban China: The mediating role of interpersonal needs. Front. Public Health.

[25] Ma L., Li Y. (2021). Intermediary role of social participation on relationship between daily life ability and mental health status in elderly people. Chin. J. Public Health.

[26] Luo W., Cheng P. (2023). Impact of Social Participation of Empty Nesters on Their Health. Med. Soc..

[27] Ma X. (2021). Social participation and self-reported health in China: Evidence from Chinese middle-aged and elderly adults. Int. J. Soc. Econ..

[28] He Q., Cui Y., Liang L., Zhong Q., Li J., Li Y., Lv X., Huang F. (2017). Social participation, willingness and quality of life: A population-based study among older adults in rural areas of China. Geriatr. Gerontol. Int..

[29] Rochelle T.L. (2023). Social participation, loneliness and well-being among older adults in Hong Kong: A longitudinal examination. Psychol. Health Med..

[30] Ejiri M., Kawai H., Fujiwara Y., Ihara K., Watanabe Y., Hirano H., Kim H.K., Ishii K., Oka K., Obuchi S. (2019). Social participation reduces isolation among Japanese older people in urban area: A 3-year longitudinal study. PLoS ONE.

[31] Sun S., Wang Y., Wang L., Lu J., Li H., Zhu J., Qian S., Zhu L., Xu H. (2024). Social anxiety and loneliness among older adults: A moderated mediation model. BMC Public Health.

[32] Dogra S., Dunstan D.W., Sugiyama T., Stathi A., Gardiner P.A., Owen N. (2022). Active Aging and Public Health: Evidence, Implications, and Opportunities. Annu. Rev. Public Health.

